# Manipulating Interfacial Stability Via Absorption-Competition Mechanism for Long-Lifespan Zn Anode

**DOI:** 10.1007/s40820-021-00777-2

**Published:** 2021-12-13

**Authors:** Meijia Qiu, Liang Ma, Peng Sun, Zilong Wang, Guofeng Cui, Wenjie Mai

**Affiliations:** 1 Key Laboratory for Polymeric Composite & Functional Materials of Ministry of Education, School of Chemistry, Key Laboratory of Low-Carbon Chemistry & Energy Conservation of Guangdong Province, Sun Yat-Sen University, Guangzhou, 510275 People’s Republic of China; 2 Siyuan Laboratory, Guangdong Provincial Engineering Technology Research Center of Vacuum Coating Technologies and New Energy Materials, Guangzhou Key Laboratory of Vacuum Coating Technologies and New Energy Materials, Department of Physics, Jinan University, Guangdong, 510632 People’s Republic of China

**Keywords:** Aromatic aldehyde, Absorption-competition mechanism, Zn anode, Interfacial stability

## Abstract

The stability of Zn anode in various Zn-based energy storage devices is the key problem to be solved. Herein, aromatic aldehyde additives are selected to modulate the interface reactions between the Zn anode and electrolyte. Through comprehensively considering electrochemical measurements, DFT calculations and FEA simulations, novel mechanisms of one kind of aromatic aldehyde, veratraldehyde in inhibiting Zn dendrite/by-products can be obtained. This additive prefers to absorb on the Zn surface than H_2_O molecules and Zn^2+^, while competes with hydrogen evolution reaction and Zn plating/stripping process via redox reactions, thus preventing the decomposition of active H_2_O near the interface and uncontrollable Zn dendrite growth via a synactic absorption-competition mechanism. As a result, Zn–Zn symmetric cells with the veratraldehyde additive realize an excellent cycling life of 3200 h under 1 mA cm^−2^/1 mAh cm^−2^ and over 800 h even under 5 mA cm^−2^/5 mAh cm^−2^. Moreover, Zn–Ti and Zn–MnO_2_ cells with the veratraldehyde additive both obtain elevated performance than that with pure ZnSO_4_ electrolyte. Finally, two more aromatic aldehyde additives are chosen to prove their universality in stabilizing Zn anodes.

**Supplementary Information:**

The online version contains supplementary material available at 10.1007/s40820-021-00777-2.

## Highlights

- Aromatic aldehyde additives were introduced to modify the interfacial environment between Zn sheet and electrolyte through an absorption-competition mechanism, thus effectively inhibiting the dendrite/by-products growth.
- Zn–Zn and Zn–MnO_2_ cells with the additives achieved much better cycling stability (over 3000 h under 1 mA cm^−2^/1 mAh cm^−2^ and 800 h under 5 mA cm^−2^/5 mAh cm^−2^ for symmetric cells) than that of pure ZnSO_4_ electrolyte.

**Supplementary Information:**

The online version contains supplementary material available at 10.1007/s40820-021-00777-2.

## Graphical abstract

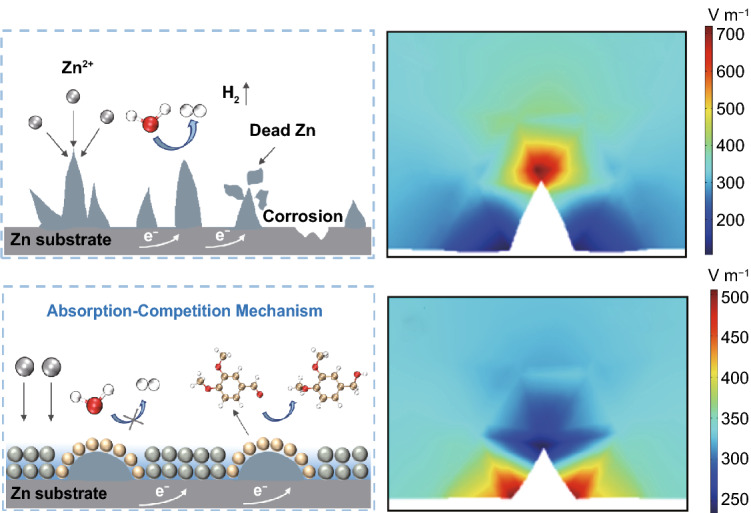

## Introduction

The strong demands toward environmentally friendly and sustainable energy storage devices have propelled the fast development of aqueous batteries [1]. Zn metal due to its proper potential (− 0.76 V vs. SHE), high volumetric capacity (5855 mAh cm^−3^) and low-cost, has been applied in all kinds of Zn-based aqueous batteries with considerable performance such as Zn ion, Ni–Zn, Ag–Zn and electrolytic Zn–MnO_2_ batteries [2–5]. The key issues inside Zn-based energy storage devices lie in the complex interface chemistry reaction easily causing partial failure of Zn metal anodes, thus usually resulting in dendrites, corrosion and by-products problems [6]. To tackle the above matters are of great significance to further progress of Zn-based devices and deeper understanding for their intrinsic mechanisms.

Great efforts on solving the severe problems in Zn metal anodes have been proposed by previous researches, most of which involved modification on Zn anode/electrolyte interface and the solvation structure of electrolyte [7–9]. For instance, strategies like decorating protective layers/solid electrolyte interphase [10–19], selecting proper separators, constructing three-dimensional alloy structure or regulating crystal orientation could effectively control the Zn ion diffusion and deposition rate [18, 20–27], while cut off the pathway of hydrogen evolution reaction (HER), random corrosion and by-products formation to some extent. Besides, another type of important methods focused mainly on designing the composition or state of the electrolyte to change the primary solvation shell constitution or the environment in Zn anode/electrolyte interface, achieving similar functions mentioned before, including introducing different solvent into pure water or small amount of additives [28–33], constructing highly concentrated “water in salt” structure and utilizing ionic liquid, eutectic liquid, hydrogels or solid-state electrolyte [34–38].

The electrolyte is one of the most important components in Zn-based energy storage devices due to the bridge function of connecting the cathode and anode while providing a pathway for Zn ions migration [39]. Through choosing proper additives into main Zn salts is a simple, cheap and easy to batch method. Moreover, the complex chemical interaction between additives with the bulk electrolyte or Zn anode surface is deserved to be explored for deeper understanding [40]. In traditional electroplating industry, there exists some brighteners achieving polished deposition via a redox mechanism, which should also be suitable for reversible Zn plating/stripping process inside all kinds of Zn-based aqueous batteries. It’s obvious a charming idea to adopt them as additives into pure electrolytes for suppressing the occurrence of dendrite/by-reactions.

We hereby proposed a type of aromatic aldehyde as additives in traditional ZnSO_4_ electrolyte to greatly elevate the stability of Zn plating/stripping in Zn ion batteries by restricting the dendrite growth and all kinds of by-reactions through an absorption-competition mechanism. Combing density functional theory (DFT) calculations and corresponding electrochemical experiments, it can be conferred the aromatic aldehyde additives preferred absorbed onto Zn metal that are capable of inhibiting common by-reactions while encountering reduction before HER and Zn deposition process at the initial nuclear process, together achieving long-lifespan Zn anode. As a proof of concept, the Zn-Zn symmetric cell, Zn-Ti and Zn-MnO_2_ devices under different electrolyte systems were fabricated and compared, confirming those aromatic aldehyde additives owned the ability to relieve the dendrite/by-products issues in Zn anodes.

## Experimental Section

### Fabrication of Zn–Zn Symmetric Cell and Zn–Ti Cells

A pair of Zn foils of 100 μm were used for constructing the symmetric cells. Different electrolyte composition (pure for 1 M ZnSO_4_ and ZnSO_4_-veratraldehyde for 1 M ZnSO_4_ with different concentration of veratraldehyde) each with 75 μL were added into the coin cell separated via a piece of glass fiber (GE-Whatman, 125 mm). As for Zn-Ti cells, we just changed the two Zn foils as one Zn foil as anode and one Ti foil as cathode, keeping the other condition same as Zn-Zn cells.

### Fabrication of Zn–MnO_2_ Full Cell

MnO_2_ was prepared on carbon cloth through electrodeposition method following our previous work [33, 41]. Firstly, carbon cloth was washed with acetone, ethanol and deionized water each for 10 min under ultrasonic bath before the electrodeposition process. Typical, a plating solution containing 0.1 M MnAc_2_ and 0.1 M Na_2_SO_4_ was used to synthesize MnO_2_ in a three-electrode system with the Ag/AgCl as reference and a carbon rod as counter electrodes. The electrodeposition condition was set to be 4 mA cm^−2^ for 10 min.

### Electrochemical Test

The performances of Zn–Zn, Zn–Ti cells and Zn–MnO_2_ full cells were collected by battery test system (Neware BTS-4000). For Zn–Zn cells, constant current densities were applied ranging from 1, 2, 5 mA cm^−2^, and the charging and discharging times were both set to be 1 h. For Zn–Ti cells, a reversible testing condition was set to be 1 mA cm^−2^ with a charging and discharging time each for 0.5 h. For Zn–MnO_2_ cells, the voltage range was 1–1.9 V under different current densities (range from 0.1 to 1 A g^−1^). The corrosion, diffusion and hydrogen evolution behaviors of Zn foil anodes in different electrochemical environments were both studied through an electrochemical workstation (CHI 660e) with a three-electrode system (Zn foil as work electrode, Pt as counter electrode and Ag/AgCl as reference electrode). The corrosion Tafel plot was recorded by performing linear voltammetry scanning (LSV) with a potential range of ± 0.3 V versus open-circle potential of the system at a scan rate of 1 mV s^−1^. The diffusion curves were collected by chronoamperometry method at an overpotential of −150 mV. The hydrogen evolution performance was collected through LSV with a potential range of −0.9 ~ −1.6 V versus Ag/AgCl at a scan rate of 1 mV s^−1^.

### Characterization

The in-situ microscope images for Zn deposition process were obtained by commercial high-resolution camera (Mshot, MS60) equipped a magnifying glass holder, the same as our previous work [33]. The morphology of Zn foil before and after deposition or cycles were characterized by field-emission scanning electron microscopy (SEM, ZEISS ULTRA 55). The crystal structure and material composition information were gathered by X-ray diffraction (XRD, Rigaku, MiniFlex600, Cu Kα).

### DFT Calculations

#### Quantum Chemistry Calculations

DFT calculation were conducted in Gaussian (G09) program [42]. The structure optimization was performed at B3LYP with 6–31 g* basis set. Then a single-point energy calculation of each optimized structure was performed at the same hybrid functional with 6311 g* basis set. The electrostatic potential (ESP) was analyzed by Multiwfn package and VMD package [43, 44].

#### Ab-Initial Calculations

The calculation related to the interaction between Zn crystal and molecules were performed by using the Vienna ab-initio Simulation Package (VASP). The projector augmented wave (PAW) potential was employed to represent the interactions of electrons with ion cores. The generalized gradient approximation (GGA) parameterized by the Perdew-Burke-Ernzerhof (PBE) method with D3 correction that was used to describe the system [45]. Implicit solvent model (H_2_O around) was also introduced in all absorption energy calculations. All calculations including geometry optimization, single-point energy and electronic density were carried out within a 13.3245 × 11.5393 × 22.4202 Å^3^ box under a periodic boundary condition and a dense Monkhorst–Pack k point mesh of 2 × 2 × 1. A high energy cutoff of 500 eV and the forces acting on all the ions smaller than 0.01 eV Å^−1^ were set. On the Z direction, there is 15 Å vacuum for erasing the effect of periodic condition for slab model. A 5 × 5 supercell with four-layer Zn slab (002) was used to represent the absorbed surface for molecules, and the bottom two layers were kept fixed to maintain bulk property. The charge differential density was calculated through the software Vesta. The specific tool Vaspkit was used for the Free-energy correction in calculating several ∆G values [46]. The absorbed energy between Zn slab and different molecules was defined as following equation:

1 $$ {\text{E}}_{{{\text{aborb}}{.}}} = {\text{ E}}_{{{\text{Zn{-}slab}} + {\text{molecules}}}} - {\text{ E}}_{{\text{Zn{-}slab}}} - {\text{ E}}_{{{\text{molecules}}}} $$

### Finite Element Analysis Method

The simplified 2D model geometry consists of two electrodes and an intermediate electrolyte domain. This simulation uses the electric field intensity and currents to present the difference between two electrolyte system models. The variable for the normal current density defines the mesh velocity. The positive electrode has some protrusions, representing uneven deposition of Zn. Before and after covered by aldehyde molecules, the electronic conductivity of the protrusions is set to change drastically.

## Results and Discussion

### Observation of Electrolyte Engineering with Veratraldehyde

**Fig. 1:**
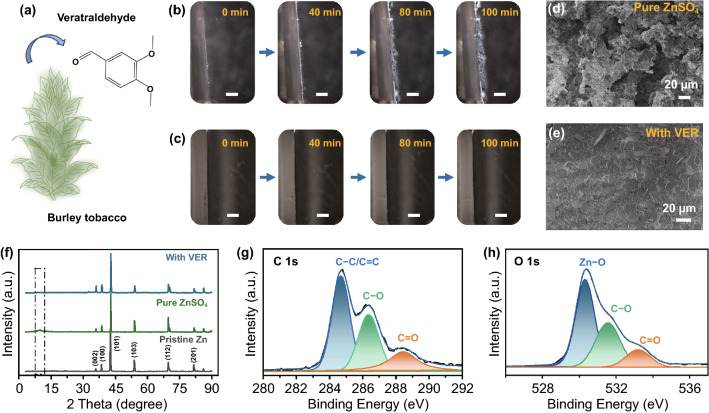
Comparison of Zn plating/stripping results under different electrolytes via morphology and constituent characterization. **a** Scheme represents one of the most common source of veratraldehyde and its molecular structure; In-situ recording microscopy images of Zn deposition process on the Ti sheet in a Zn//Ti system under **b** ZnSO_4_ and **c** ZnSO_4_-veratraldehyde environments, white scale bar: 200 μm; SEM images of Zn sheet surface after 50 plating/stripping cycles in Zn//Zn symmetric cells with a test condition of 5 mA cm^−2^, 5 mAh cm^−2^ under **d** ZnSO_4_, **e**ZnSO_4_-veratraldehyde electrolytes and **f** their corresponding XRD patterns. The High-resolution XPS spectra of the Zn sheet: **g** C 1s and **h** O 1s after soaked in 0.3 g L^−1^ veratraldehyde solution for a week

One of the commonly used aromatic aldehyde spice, veratraldehyde was selected first. As presented in Fig. 1a, it can be derived from a plant called burley tobacco, thus is eco-friendly and abundant in source. The intuitive function for inhibiting dendrite growth was firstly observed through in-situ optical microscopy images under a deposition condition of 5 mA cm^−2^ with pure ZnSO_4_ and ZnSO_4_-veratraldehyde electrolytes in Fig. 1b, c, respectively. Ti foils were used as the anode to deposit Zn due to their small lattice mismatch (lattice misfit equals 9%) [24]. Non-uniform growth of Zn metals or other by-products could gradually be found on the Ti foil as the plating process continued under pure ZnSO_4_ electrolyte (Movie S1). However, for the sample within an environment containing a small amount of veratraldehyde (0.3 g L^−1^), only smooth and plain deposited layer existed on the Ti foil (Movie S2). These results provide the preliminary evidence that veratraldehyde is a valid additive for stabilizing Zn anode. Subsequently, two Zn sheets were coupled into a symmetric cell for reversible plating/stripping test. After 50 cycles under a current density of 5 mA cm^−2^ and areal capacity of 5 mAh cm^−2^, their surface morphology under different electrolyte system was compared through scanning electron microscope (SEM), as demonstrated in Fig. 1d, e and S1. Prominent aggregations of uneven Zn and by-products deposition were caught everywhere in the sample experiencing cyclic deposition/dissolution in pure ZnSO_4_ electrolyte. In sharp contrast, a uniform and plain layer could be discovered on the surface of the Zn sheets encountering the same reversible plating/stripping process in a electrolyte with veratraldehyde additive. Besides, X-Ray diffraction (XRD) measurements were performed for above two kinds Zn sheets after long plating/stripping cycles under different electrolytes. The peak for common by-product (Zn(OH)_2_))_3_(ZnSO_4_)–(H_2_O)_3_ at 2*θ* = 9.7° can be easily observed in the sample under pure ZnSO_4_ electrolyte. In reverse, Zn sheets under the ZnSO_4_-veratraldehyde electrolyte exhibit totally no impurity peak but the similar peaks as pure Zn sheet. These characterizations further indicate the essential role of the veratraldehyde additive in solving the dendrite problem on Zn anodes. The absorption behavior of the veratraldehyde additive was examined through X-ray photoelectron spectroscopy (XPS) analysis. One Zn sheet was soaked into 0.3 g L^−1^ veratraldehyde solution and performed this test. From the XRD results in Fig. S2, it can be conferred that no by-products can be found on the treated Zn sheet. As presented in Fig. 1g, h, peaks located at 284.68 eV, 286.38/531.58 eV and 288.48/533.18 eV from C 1s and O 1s are corresponding to C–C/C=C of benzene ring, C–O and C=O all derived from the veratraldehyde molecule, respectively, while the peak at 530.28 eV might belong to Zn–O due to the interaction between the Zn sheet and veratraldehyde or some by-products oxidized by H_2_O/O_2_ molecules in the electrolyte/air interface (proved by the XPS result from Zn sheet soaked in DI water for a day, Fig. S2) [47–49], while the peak located at 1021.88 eV appeared a small shift toward higher binding energy in Zn 2p compared with pure Zn sheet (shown in Fig. S3), indicating that this additive can be loaded on Zn surface via a chemical absorption function.

### Interfacial Interaction Between the Zn Anode and Electrolyte

**Fig. 2:**
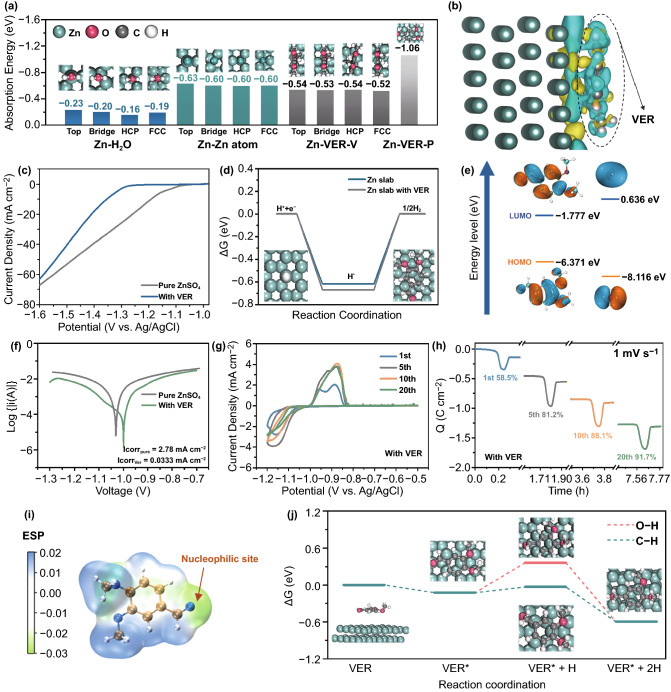
Electrochemical measurements and DFT calculations for exploiting the function of the veratraldehyde additive inside ZnSO_4_ electrolyte. **a** Absorption energy comparison of Zn atom, H_2_O and veratraldehyde molecules on Zn (002) crystal plane, insets show the corresponding absorbed models for different situations; **b** The charge density difference model of Zn slab with parallelly placed veratraldehyde and the corresponding isosurface (yellow and cyanine semi-transparent clusters represent increase and decrease of electron density, respectively); Comparison of **c** the HER performance and **d** the free-energy graphs of the HER processes between pure ZnSO_4_ and ZnSO_4_-veratraldehyde electrolytes; **e** LUMO, HOMO isosurfaces (isovalue = 0.02 a. u.) of veratraldehyde (left) and water molecules (right); **f** Comparison of Tafel plots representing the corrosion behavior under different electrolytes; **g** CV curves of Zn plating/stripping at a scan rate of 1 mV s^−1^ in the ZnSO_4_-veratraldehyde electrolyte and **h** the corresponding chronocoulometry curves based on the above CV curves; **i** Electrostatic potential mapping of one veratraldehyde molecule indicating the obvious nucleophilic site; **j** Possible reaction pathways for veratraldehyde reduced to veratryl alcohol on the surface of Zn slab

In order to figure out the exact mechanism of the veratraldehyde additive in inhibiting dendrite/by-products growth, DFT calculations combined with several electrochemical experiments were conducted. Due to the complex but important issues happened in the interface between the Zn metal and electrolyte, our research mainly pointed on their interfacial interactions. Ab-initio calculations were firstly performed to study the absorption ability of different molecules/ions on Zn metal slab-(002) crystal plane, exhibited in Fig. 2a. Various possible absorbed sites (details shown in Fig. S4) for H_2_O, veratraldehyde molecules and Zn ions were all taken into account. Results demonstrated that there existed weak interaction between H_2_O and Zn slab (the most stable site lied in TOP, the absorption energy E_abs_ only reached −0.23 eV). For the situation of Zn ions loaded on Zn slab, rather strong absorptive function could be observed, with all E_abs_ over −0.60 eV, which even exceeded those models of Zn slab with vertically aligned veratraldehyde molecules (Zn-VER-V), no matter which kinds of sites. However, when veratraldehyde was arranged on Zn slab parallelly (Zn-VER-P), it presented the highest E_abs_ (− 1.06 eV). The interaction between the Zn anode and veratraldehyde molecules was further studied through charge density difference calculations in Fig. 2b. Apparent charge transfer could be observed between the two objects, indicating a fairly strong chemical absorption behavior. In addition, electrochemical measurements including nucleation overpotential and electrochemical impedance spectroscopy (EIS) under different electrolytes were compared. As presented in Fig. S5, Zn–Zn cells using the ZnSO_4_-veratraldehyde electrolyte possessed a higher charge transfer resistance (346.1 Ω) and nucleation overpotential (93.6 mV) than that with pure ZnSO_4_ electrolyte (65.1 Ω and 50.9 mV), further proving the absorption behavior of the veratraldehyde additive. Thus, it can be inferred that the veratraldehyde molecule preferred to interact with Zn anode than the H_2_O molecule and Zn ions and help to control the Zn ion diffusion process, as also proved via the chronoamperometry measurements in Fig. S6. Under an overpotential of − 150 mV, the current density under pure ZnSO_4_ system continuously decreased without slowing the damping speed till 600 s, representing an uncontrollably rampant 2D and subsequent 3D diffusions. On the contrary, that one with the veratraldehyde additive presented mild variation of current density as time went by, and reached a steady state finally, which is in accordance with the constrained 2D diffusion behavior. Moreover, the system with the veratraldehyde additive could greatly change the onset potential of HER to negative side (over 200 mV variation), inducing larger overpotential (163 mV difference) under the current density of 10 mA cm^−2^ (Fig. 2c). Similar results could further be proved according to theoretical calculations for the free energy of hydrogen adsorption (G) on Zn (002) slab, as shown in Fig. 2d. Obviously, a higher variation of G (∆G) of − 0.67 eV under the electrolyte system with the veratraldehyde additive versus − 0.62 eV under a pure ZnSO_4_ electrolyte exhibited the better inhibition function of the veratraldehyde for the HER process, well in line with above experimental results. Comparison of molecular orbital level between the veratraldehyde and H_2_O molecules was performed afterward, as shown in Fig. 2e. The veratraldehyde delivers a lower position of the lowest unoccupied molecular orbital (LUMO) than that of H_2_O (− 1.777 vs. − 0.636 eV), implying that it is easier to obtain electron, thus effectively restraining the decomposition of H_2_O. In addition, the anti-corrosion ability was also studied through a linear voltammetry test (Fig. 2f). The introduce of veratraldehyde was capable of reducing the corrosion current from 2.78 to 0.0333 mA cm^−2^, indicating that this additive would bring effective restriction for various of side-reactions during the Zn plating/stripping process. All of these illustrated that higher capability of inhibiting the decomposition of active H_2_O molecules and growth of various by-products could be achieved when the veratraldehyde was added into the pure ZnSO_4_ electrolyte.

To get a deeper understanding in the function of the veratraldehyde additive, CV curves for reversible Zn plating/stripping measurements on Ti were collected. As can be seen in Fig. S7a, a pair of stripping/plating peaks were easily found at around − 0.80 and − 1.20 V (versus Ag/AgCl) in nearly each cycle under a pure ZnSO_4_ electrolyte. In contrast, when the veratraldehyde was introduced into the electrolyte system, there appeared one more pair of redox peaks except that for Zn plating/stripping process presented in Fig. 2g at the first five CV cycles, which should be originated from the redox process of the reversible transformation between the veratraldehyde additive and veratryl alcohol molecules (see formula in Fig. S8). It’s worth noticing that the extra pair of peaks located at around − 0.95 and − 1.15 V were all prior to the dissolution/deposition of Zn, which was helpful for slowing the Zn plating/stripping speed and resulted in constrained 2D diffusion process. Their corresponding chronocoulometry curves according to CV measurements at specific cycles were then calculated (Figs. S7b and 2 h). The coulombic efficiency (CE) under pure ZnSO_4_ electrolyte demonstrated an irregular flucturation among different cycles, all lower than 85%, while for the ZnSO_4_-veratraldehyde electrolyte system, CE value gradually increased to over 90%, indicating that the veratraldehyde additive contributed to more stable Zn plating/stripping behaviors. To figure out detailed chemical reaction of the veratraldehyde additive accompanying the main process of reversible Zn/Zn^2+^ conversion in the electrode/electrolyte interface, several DFT calculations were applied again. As depicted in Fig. 2i, the C=O bond from its aldehyde group owns the lowest electrostatic potential, thus becoming the nucleophilic site during reduction process. Furthermore, the free-energy variation of the reduction process of a veratraldehyde molecule absorbed on Zn (002) slab was studied in Fig. 2j, where two possible reaction paths all including three steps were compared. It can be inferred that the aldehyde group from the veratraldehyde molecule prefer to acquire proton on C atom and finally reach the totally reduced state-veratryl alcohol. ∆G of this reaction (− 0.47 eV) was lower than that of HER process mentioned above (∆G =  − 0.67 eV), indicating the reduction of the veratraldehyde happened in priority. It should be noted that even some veratraldehyde additives were reduced to veratryl alcohol molecules, their absorption on Zn slab is also capable of suppressing the production of H_2_ while decrease the corrosion current at the same time, as proved by the DFT calculation and electrochemical test results in Fig. S9.

**Fig. 3:**
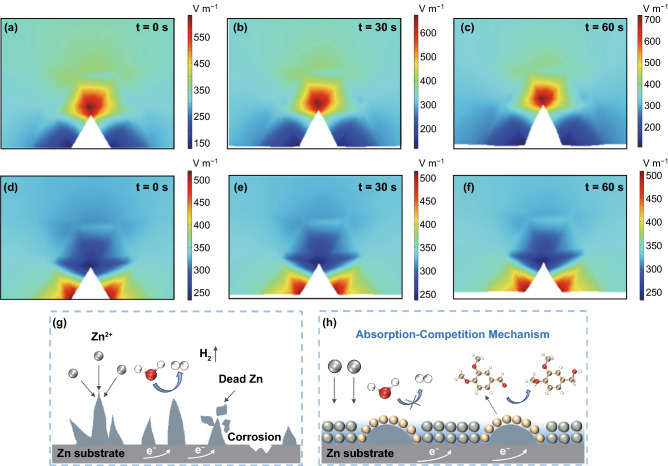
Finite Element Analysis for electric field intensity in Zn anode surface under different electrolyte systems and the scheme describing the inhibiting mechanism for dendrite growth of veratraldehyde. Models of electric field intensity distribution under **a**–**c** pure ZnSO_4_ and **d**–**f** ZnSO_4_-veratraldehyde electrolytes at different deposition time; Schemes of electrochemical reactions in Zn anode/electrolyte interface under **g** ZnSO_4_ and **h** ZnSO_4_-veratraldehyde electrolytes

After studying the insight mechanism in inhibiting Zn dendrite/by-products growth of the veratraldehyde additive in microscale (several nanometers) via DTF methods, finite element analysis (FEA) in macroscale (hundreds of micrometers) was further conducted to observe its obvious function. As demonstrated in Fig. 3a–c, the electric field intensity for the tip of one dendrite nuclear obviously exhibit much larger value than those area at the bottom space. Therefore, dendrites prefer to grow higher as the depositing time was prolonged to a period of time. Strikingly, the addition of veratraldehyde is evidently able to influence the distribution of electric field intensity, that is, badly impair their intensity in the tip of the dendrite core to lower than those at the bottom space, as presented in Fig. 3d–f. Besides, after enough deposition time (300 and 600 s), the Zn dendrite can be filled to a smaller state and leveled up finally (Fig. S10).

Concluding the above experimental and theory analysis, the comprehensive mechanism for controlling the production of Zn dendrites/by-products of the veratraldehyde additive can be described, as demonstrated in schemes of Fig. 3g, h. In traditionally pure ZnSO_4_ electrolyte, detrimental H_2_O molecules distributed in the Zn anode interface can be easily ionized into H^+^ and OH^−^. On the Zn surface with abundant electrons, aggregated H^+^ ions are easily reduced to H_2_ and stay away from the electrolyte system, leaving much OH^−^ and increasing the pH vales. These usually lead to rampant side-reactions including corrosion of Zn metal and generation of common by-products. Besides, the deposition process of Zn^2+^ is violent and uncontrollable, resulting in uneven growth of Zn dendrite and even breaking away from Zn anode into dead Zn. The introduced veratraldehyde additive is inclined to absorb on Zn anode surface before H_2_O molecules and Zn^2+^ ions, thus achieving the following effects: (1) It can effectively inhibit the HER process, thus retaining the pH of electrolyte in a proper value and suppressing the occurrence of all kinds of side-reactions; (2) Redox reactions between the veratraldehyde and veratryl alcohol are prior to the Zn stripping/plating process, which is helpful for controlling the rampant 2D diffusion process and bringing uniform deposition/dissolution of Zn through a competition behavior.

### All Kinds of Cells Evaluation

**Fig. 4:**
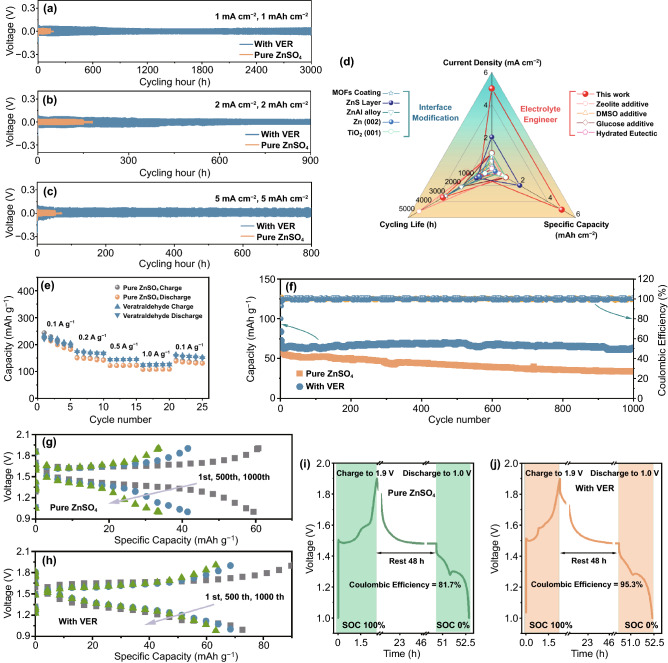
Performance comparison of Zn–Zn, Zn–Ti and Zn–MnO_2_ cells under different electrolyte environments. Long-term cycling performance comparison of Zn–Zn symmetric cells under different current densities and areal capacities of **a** 1 mA cm^−2^, 1 mAh cm^−2^, **b** 2 mA cm^−2^, 2 mAh cm^−2^ and **c** 5 mA cm^−2^, 5 mAh cm^−2^ in different electrolytes; **d** performance comparison of Zn–Zn symmetric cells between this work and previous work; **e** rate capacities at different current densities ranging from 0.1 to 1.0 A g^−1^, **f** cycling stability at a current density of 3.08 A g^−1^ and **g**, **h** corresponding galvanostatic curves of Zn–MnO_2_ full cells under different electrolyte systems; Self-discharge performance of Zn–MnO_2_ full cells under **i** pure ZnSO_4_ and **j** ZnSO_4_-veratraldehyde electrolytes

To further affirm the attracting function of the veratraldehyde additive in stabilizing Zn anodes during reversible plating/stripping reactions, Zn–Zn, Zn–Ti and Zn–MnO_2_ cells were all fabricated to study its effect in actual energy storage devices. Firstly, cycling stability of Zn–Zn symmetric cells in several current densities and deposited capacities with different electrolyte systems were compared, as presented in Fig. 4a–c. In pure ZnSO_4_ electrolyte, under the testing condition of 1 mA cm^−2^/1 mAh cm^−2^ and 2 mA cm^−2^/2 mAh cm^−2^, the symmetric cells encountered short circuit induced by developed Zn dendrite all at around 150 h. After the addition of the veratraldehyde additive, Zn–Zn cells can achieve a super-long cycling life of over 3200 h, 900 h under 1 mA cm^−2^/1 mAh cm^−2^, 2 mA cm^−2^/2 mAh cm^−2^, respectively, and great stability (over 800 h) even at a high current density/capacity of 5 mAcm^−2^/5 mAh cm^−2^, much better than that with pure ZnSO_4_ electrolyte (54 h) and most of the previous work (Fig. 4d) [12, 14, 20, 23, 32, 33, 35, 50, 51]. Moreover, the veratraldehyde also can ensure stable rate cycling performance in Zn–Zn cells (Fig. S11a) under conditions ranging from 1 mA cm^−2^/1 mAh cm^−2^, 2 mA cm^−2^/2 mAh cm^−2^, 5 mAcm^−2^/5 mAh cm^−2^ and finally back to 1 mA cm^−2^/1 mAh cm^−2^ each for 100 h, distinctly better than that of pure ZnSO_4_ electrolyte (Fig. S11b). Proper concentration of the veratraldehyde additive was further studied, as demonstrated in Fig. S12. Gradually increasing amount of veratraldehyde (before 0.3 g L^−1^) can effectively boost the cycling stability of Zn–Zn cells. After the concentration was further increased to 0.4 g L^−1^ (close to its solubility limit), no obvious enhancement in stability could be observed, but resulting a larger polarization voltage. Therefore, 0.3 g L^−1^ was selected as the final concentration. It is worth noting that Zn–Zn cells under the ZnSO_4_ electrolyte with a veratryl alcohol additive can also achieve great performance (over 320 h at 5 mAcm^−2^/5 mAh cm^−2^, as shown in Fig. S13), demonstrating that even though some veratraldehyde molecules were reduced to veratryl alcohol during long-term test, the Zn anode could still be well protected both by the rest veratraldehyde and new formed veratryl alcohol. Subsquently, Zn plating/stripping coulombic efficiency (CE) was evaluated via constructing Zn–Ti cells under different electrolytes. As exhibited in Fig. S14, at first 80 cycles, Zn–Ti cells with pure ZnSO_4_ electrolyte possess stable CE and then appears fluctuate as a result of the uncontrollable growth of dendrite or several by-products. However for veratraldehyde-ZnSO_4_ environment, CE of Zn–Ti cells continued to increase and all reached over 97% after 100 cycles, indicating excellent repellency for all kinds of side-reactions and fierce dendrite growth.

The Zn–MnO_2_ full cells were finally assembled through combining electrodeposited MnO_2_ on carbon cloth and Zn foil to study the practical availability in energy storage devices of the veratraldehyde additive. It can be clearly found that the introduction of the veratraldehyde additive is capable of elevating overall performance of full cells. As indicated in Figs. 4e and S15, the rate performance and corresponding galvanostatic charge–discharge (GCD) curves at a current density of 0.1 A g^−1^ comparisons were collected. Obviously, devices with the veratraldehyde additive performed comparable mass specific capacity than that of pure ZnSO_4_ electrolyte under different current densities, implying the addition of veratraldehyde hardly influence the energy storage ability of practical devices. CV cures of the Zn-MnO_2_ full cells with a veratraldehyde-ZnSO_4_ electrolyte under different scan rates showed two pair of redox peaks, which was related to the two-steps Zn^2+^ insertion/extraction process for the MnO_2_ cathode (Fig. S16). As for cycling stability (shown in Fig. 4f–h), the full cells with the veratraldehyde additive achieved much better performance than that of pure ZnSO_4_ electrolyte, with a capacity retention of 75.4% over 45.5% after 1000 GCD cycles at a current density of 1 A g^−1^. The prevention ability for parasitic reactions of the veratraldehyde additive inside full cells can also be proved via the GCD and self-discharge measurements. Zn-MnO_2_ devices with different electrolyte systems were all charged to 1.9 V, subsquently rested for 48 h and finally discharged to 1 V (Fig. 4i, j). There existed remarkable difference in CE value with different electrolytes (95.3% for veratraldehyde-ZnSO_4_ and 81.7% for pure ZnSO_4_), demonstrating the excellent function of the veratraldehyde in restraining all kinds of side-reactions.

### Universality of Aromatic Aldehyde-Based Additive for Stabilizing Zn Anode

**Fig. 5:**
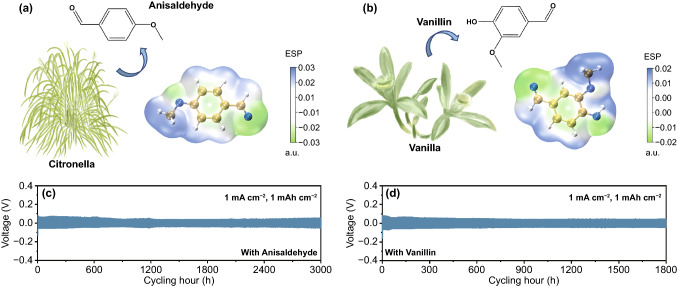
Universal application of different aromatic aldehydes as additive for suppressing Zn dendrite. Schemes indicating two commonly used aromatic aldehydes **a** anisaldehyde, **b** vanillin and their corresponding molecular structure, electrostatic potential mapping; Long-term cycling performances of Zn–Zn symmetric cells at a current density and areal capacity of 1 mA cm^−2^, 1 mAh cm^−2^ under **c** ZnSO_4_-anisaldehyde and **d** ZnSO_4_-vanillin electrolytes

At last, for confirming the universal effect of aromatic aldehyde type additives for obtaining stable Zn plating/stripping process, two more kinds of aromatic aldehyde, anisaldehyde (usually found in citronella) and vanillin (usually found in vanilla) were selected. As demonstrated in Fig. 5a, b, their electrostatic potential mappings were firstly calculated, indicating their nucleophilic sites still located at the C–O bond from the aldehyde group, the same as the veratraldehyde. As a proof of concept, Zn–Zn symmetric cells with these two additives were constructed, and their reversible plating/stripping stability were studied, as presented in Figs. 5c, d and S17. For anisaldehyde-ZnSO_4_ system, the symmetric cells reached over 3000 h and 200 h cycles for testing conditions of 1 mA cm^−2^/1 mAh cm^−2^ and 5 mA cm^−2^/5mAh cm^−2^, respectively, while for the vanillin-ZnSO_4_ one, 1800 h and 300 h cycles under measurement conditions of 1 mA cm^−2^/1 mAh cm^−2^ and 5 mA cm^−2^/5mAh cm^−2^ were achieved, all much better than that of pure ZnSO_4_ system.

## Conclusions

In summary, we proposed some aromatic aldehyde molecules as a kind of electrolyte additives in traditional ZnSO_4_ electrolyte for Zn ion batteries and achieved great performance in stabilizing Zn anodes. Choosing one of the most common aromatic aldehydes, veratraldehyde as an example, unique realization mechanism in inhibiting the growth of Zn dendrite/by-products was explored. Combing various electrochemical measurements and DFT calculations, it can be inferred that the veratraldehyde is prone to absorb on the Zn surface than H_2_O and Zn^2+^, preventing the conventional HER reactions derived from the decomposition of active H_2_O molecules around the Zn anode interface and uncontrollable deposition of Zn^2+^. Furtherly, veratraldehyde molecules owned a lower redox potential than that of Zn plating/stripping, which efficiently stabilized the initial nucleation process of deposited Zn layer and further limited those side-reactions including HER, corrosion and increase of dead Zn metal via a competing process. Macroscopic FEA simulations indicated that the absorption of the veratraldehyde is capable of impairing the electric field intensity in the tip of dendrite nuclear, thus resulting a final plain plating layer. As a prove of concept, Zn–Zn symmetric cells with a veratraldehyde additive can reach a super-long cycling life of 3200 h at a current density/capacity of 1 mA cm^−2^/1 mAh cm^−2^, and over 800 h at 5 mA cm^−2^/5 mAh cm^−2^, far more than those of with pure ZnSO_4_ electrolyte (150 and 54 h, respectively). Besides, Zn–Ti cell using ZnSO_4_-veratraldehyde electrolyte demonstrated great CE (over 97% for over 200 cycles), obviously more stable than that of pure ZnSO_4_ system. Furtherly, Zn–MnO_2_ full cells under a ZnSO_4_-veratraldehyde environment exhibited much better cycling stability and self-discharge CE, implying the excellent function of the veratraldehyde in stabilizing the Zn anode and whole practical cells. Finally, two more other aromatic aldehyde, anisaldehyde and vanillin were chosen to prove their universality as additives for stable Zn anode in Zn ion batteries. Zn–Zn symmetric cells using above two additives all realized outstanding cycling stability at several measuring conditions, indicating the aromatic aldehyde-based additives could be one of the attractive candidates in future electrolyte engineer design and commercial use of all kinds of Zn-based energy storage devices.

## Supplementary Information

Below is the link to the electronic supplementary material.

Supplementary file1 (PDF 1676 kb)

Supplementary file2 (GIF 8992 kb)

Supplementary file3 (GIF 4757 kb)
