# Supplementary material for: Manipulating Interfacial Stability Via Absorption-Competition Mechanism for Long-Lifespan Zn Anode
Source: Nanomicro Lett. 2021 Dec 13;14:31. doi: 10.1007/s40820-021-00777-2 (PMC8669073; doi:10.1007/s40820-021-00777-2)
Supplement: Supplementary file 1 — Supplementary file1 (PDF 1676 kb) [file 40820_2021_777_MOESM1_ESM.pdf]

Supporting Information for

Manipulating Interfacial Stability *via* Absorption-Competition Mechanism for Long-Lifespan Zn Anode

Meijia Qiu[1, 2], Liang Ma[2], Peng Sun[1, 2], Zilong Wang[2], Guofeng Cui[1, *], Wenjie Mai[2]

[1]Key Laboratory for Polymeric Composite & Functional Materials of Ministry of Education, School of Chemistry, Key Laboratory of Low-carbon Chemistry & Energy Conservation of Guangdong Province, Sun Yat-sen University, Guangzhou 510275, People's Republic of China

[2]Siyuan Laboratory, Guangdong Provincial Engineering Technology Research Center of Vacuum Coating Technologies and New Energy Materials, Guangzhou Key Laboratory of Vacuum Coating Technologies and New Energy Materials, Department of Physics, Jinan University, Guangdong 510632, People's Republic of China

*Corresponding author. E-mail: cuigf@mail.sysu.edu.cn (Guofeng Cui)

Supplementary Figures

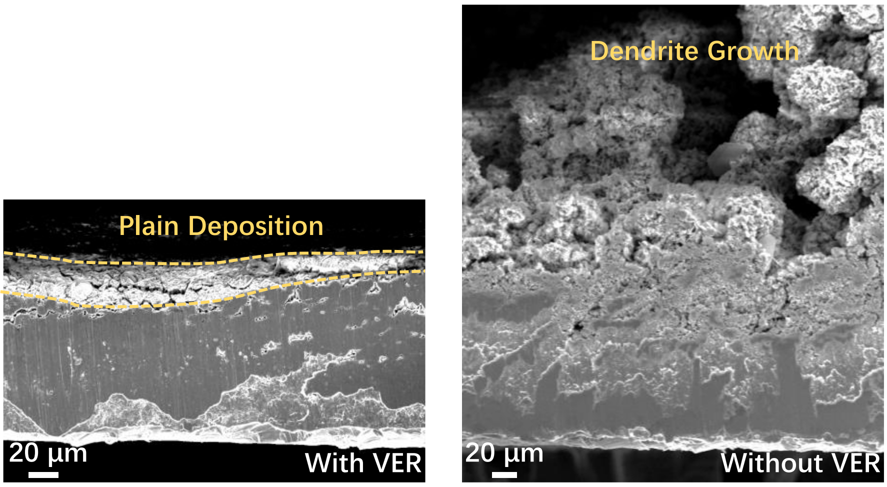

Fig. S1 Cross-sectional SEM images of Zn sheet surface with (left) and without (right) the veratraldehyde additive

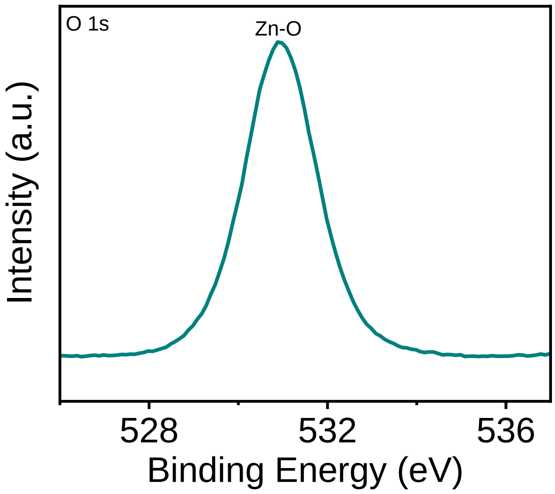

Fig. S2 The high-resolution XPS spectra of O 1s for the Zn sheet after soaked in DI water

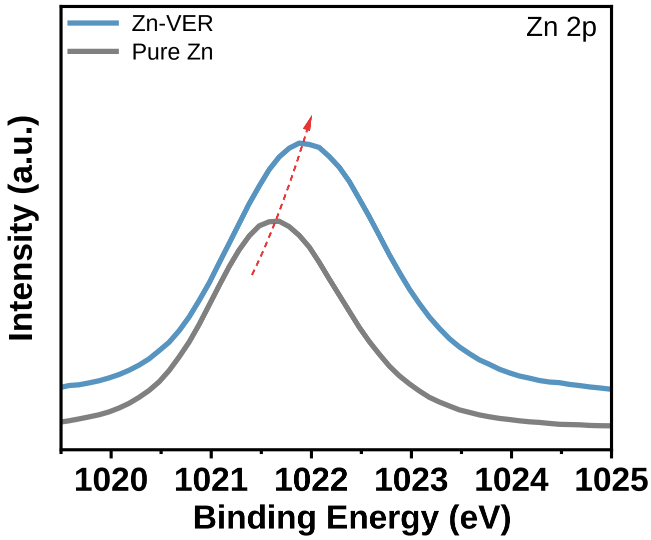

Fig. S3 Comparison of the high-resolution XPS spectra of Zn 2p between pure Zn sheet and the Zn sheet after soaked in 0.3 g L^{-1} veratraldehyde solution

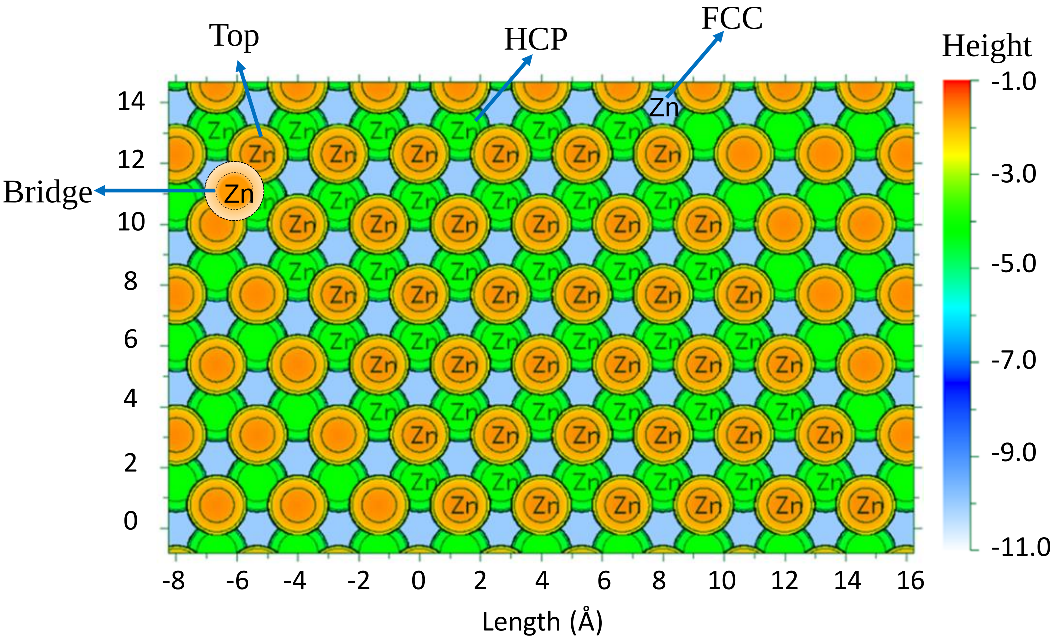

Fig. S4 Top view of the Zn slab with possible absorbed sites

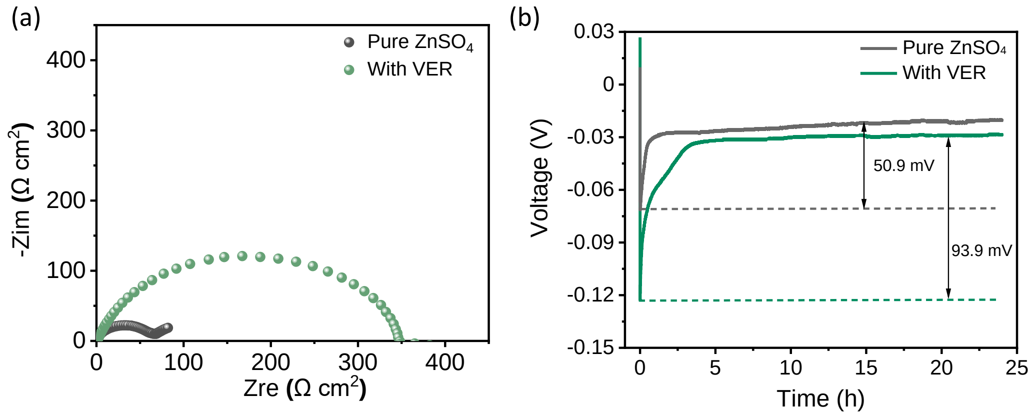

Fig. S5 (**a**) EIS and (**b**) nucleation overpotential comparison of Zn-Zn symmetric cells under electrolyte systems with and without veratraldehyde additive

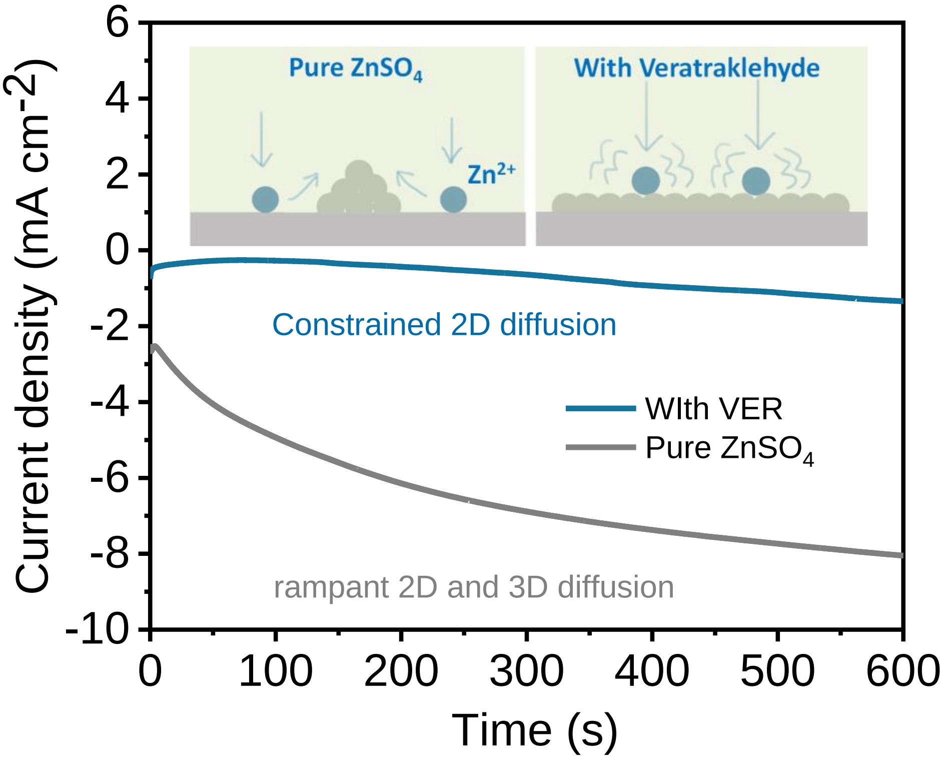

Fig. S6 Chronoamperometry curves indicating Zn^{2+} diffusion process of Zn electrode under different electrolyte system

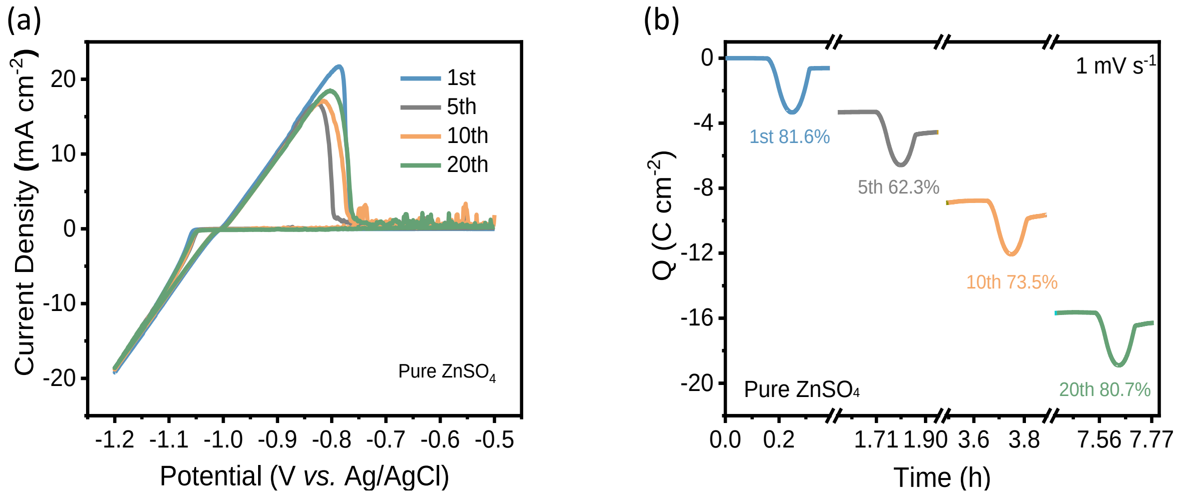

Fig. S7 (**a**) CV curves of Zn plating/stripping at a scan rate of 1 mV s^{-1} in the pure $ZnSO_4$ electrolyte and (**b**) corresponding chronocoulometry curves based on above CV curves

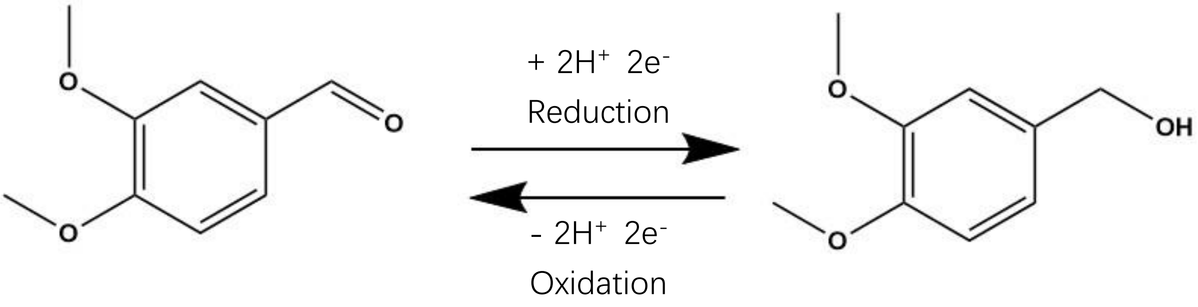

Fig. S8 Reaction formula of reversible redox process between the veratraldehyde and veratryl alcohol molecules

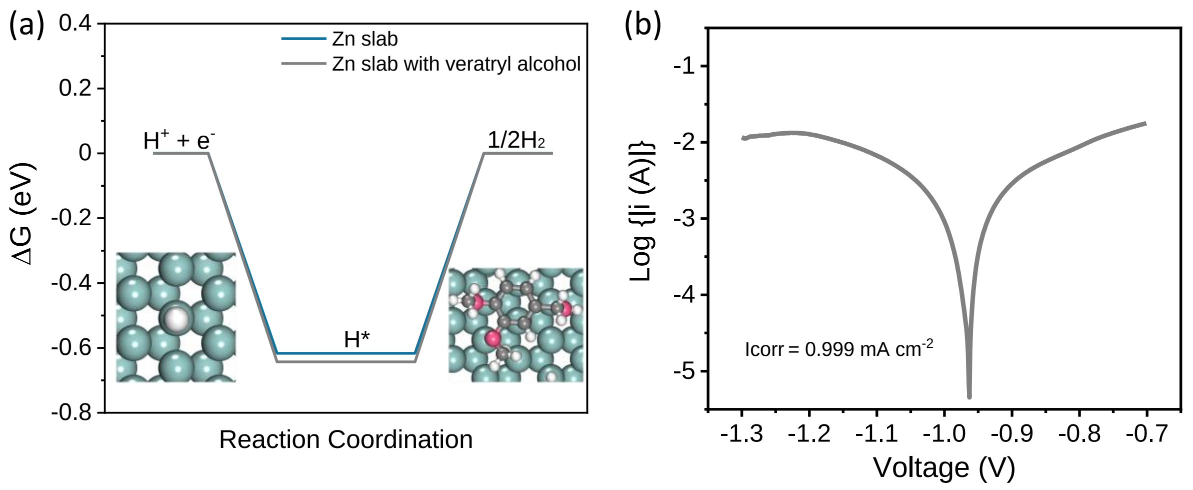

Fig. S9 (**a**) The HER diagrams of pure Zn slab and Zn slab with absorbed veratryl alcohol; (**b**) Tafel plot representing the corrosion behavior under ZnSO4-veratryl alcohol electrolyte

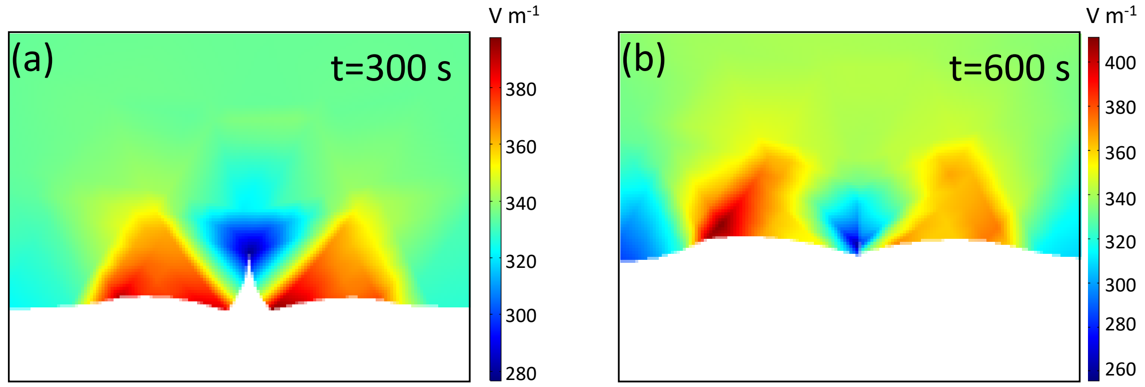

Fig. S10 Models of electric field intensity distribution under (**a**)-(**b**) $ZnSO_4$-veratraldehyde electrolytes at different reaction time

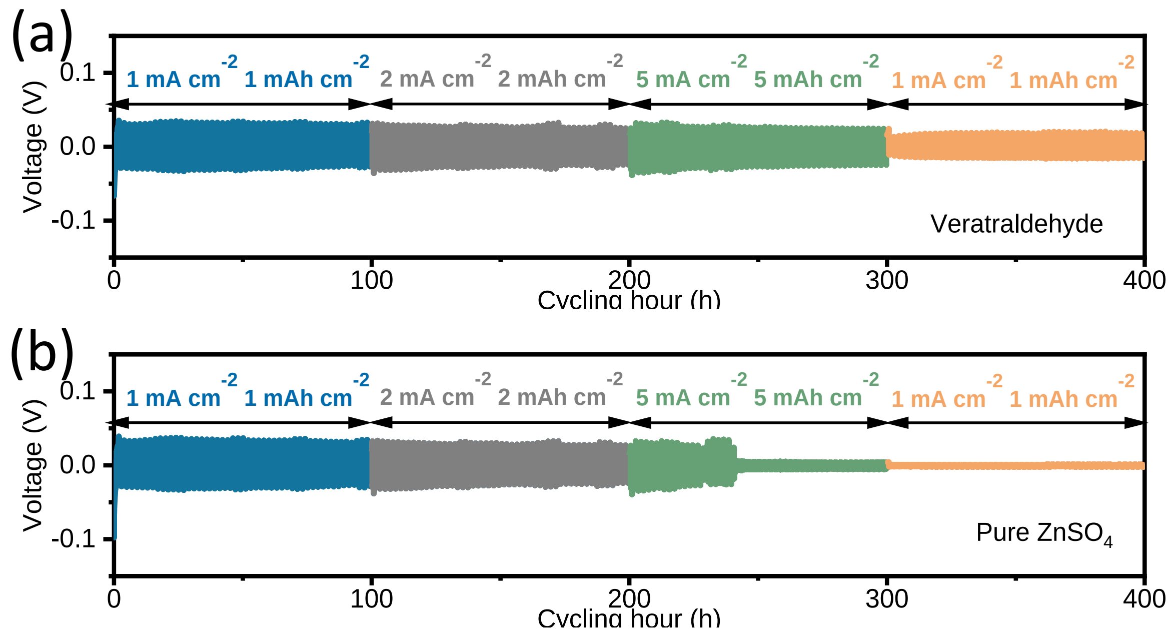

Fig. S11 Rate cycling performance comparison of Zn-Zn symmetric cells under different current densities and areal capacities of 1 mA cm^{-2}, 1 mAh cm^{-2}; 2 mA cm^{-2}, 2 mAh cm^{-2} and 5 mA cm^{-2}, 5 mAh cm^{-2} in (**a**) $ZnSO_4$-veratraldehyde and (**b**) pure $ZnSO_4$ electrolytes

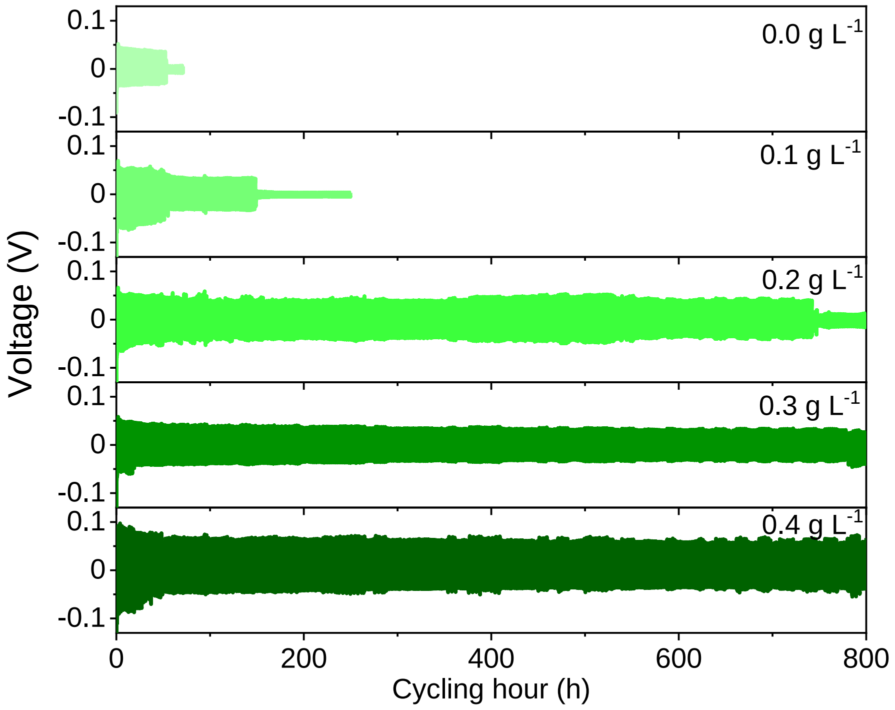

Fig. S12 Long-term cycling performance comparison of Zn-Zn symmetric cells under current densities and areal capacities of 5 mA cm^{-2}, 5 mAh cm^{-2} in $ZnSO_4$ electrolytes with different veratraldehyde concentration

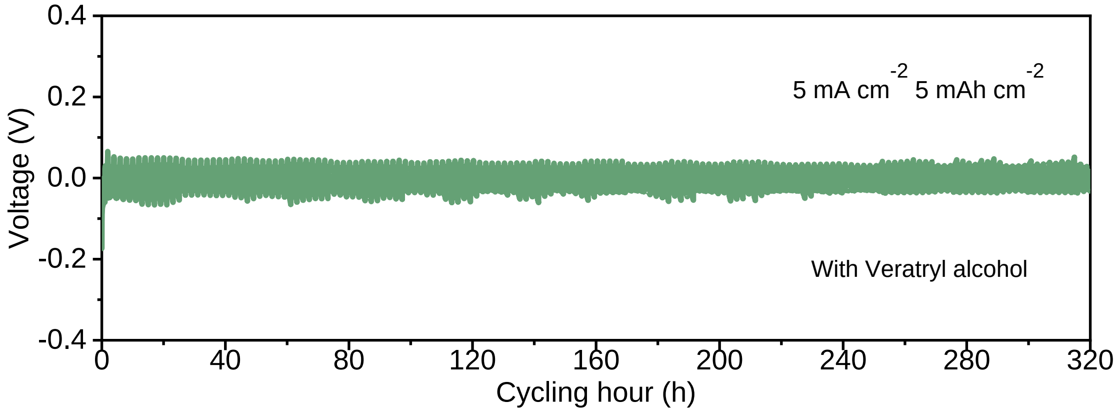

Fig. S13 Long-term cycling performance comparison of Zn-Zn symmetric cells under current densities and areal capacities of 5 mA cm^{-2}, 5 mAh cm^{-2} in $ZnSO_4$ electrolytes with alcohol additive

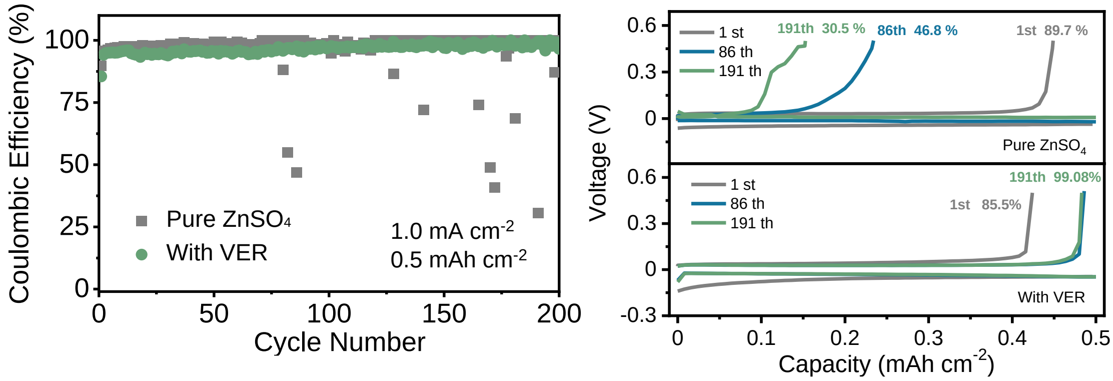

Fig. S14 Coulombic efficiency (CE) measurements of Zn//Ti cells and corresponding voltage profiles at various cycles under pure $ZnSO_4$ and $ZnSO_4$-veratraldehyde electrolytes

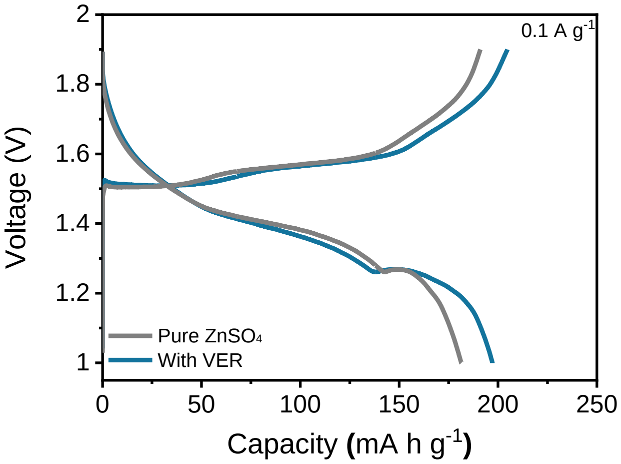

Fig. S15 GCD curves at a current density of 0.1 A g^{-1} in different electrolytes

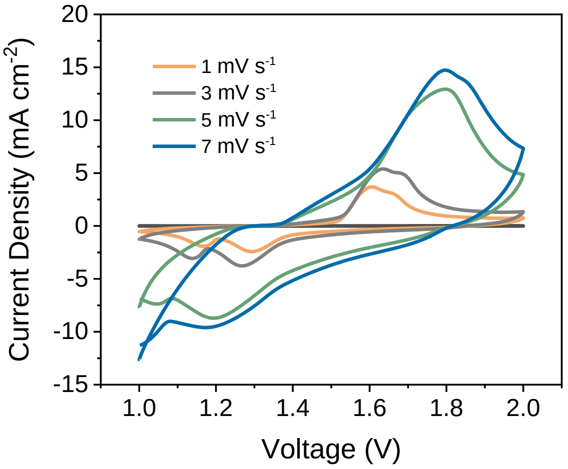

Fig. S16 CV curves of Zn-MnO_2 batteries under different scan rates ranging from 1 mV s^{-1} to 7 mV s^{-1}

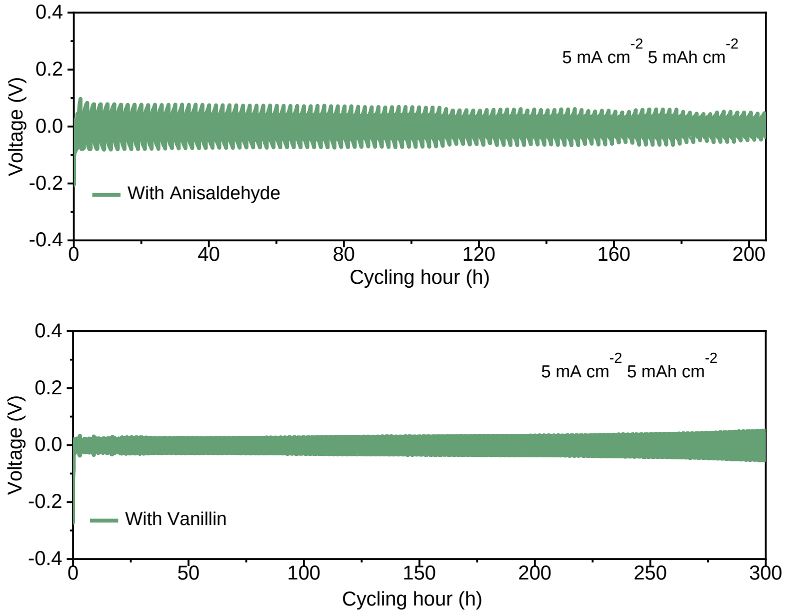

Fig. S17 Long-term cycling performance comparison of Zn-Zn symmetric cells under current densities and areal capacities of 5 mA cm^{-2}, 5 mAh cm^{-2} in $ZnSO_4$ electrolytes with anisaldehyde and vanillin additives
