# Supplementary figures and images for: Manipulating Interfacial Stability Via Absorption-Competition Mechanism for Long-Lifespan Zn Anode
Source: Nanomicro Lett. 2021 Dec 13;14:31. doi: 10.1007/s40820-021-00777-2 (PMC8669073; doi:10.1007/s40820-021-00777-2)

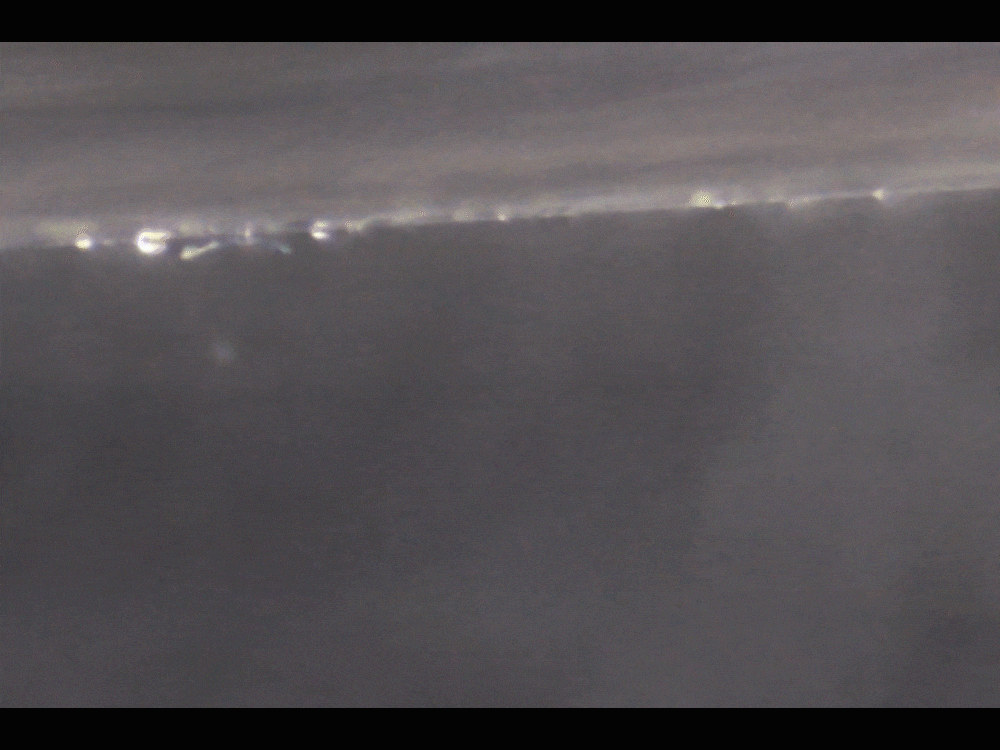

Supplement: Supplementary file 2 — Supplementary file2 (GIF 8992 kb) [file 40820_2021_777_MOESM2_ESM.gif]

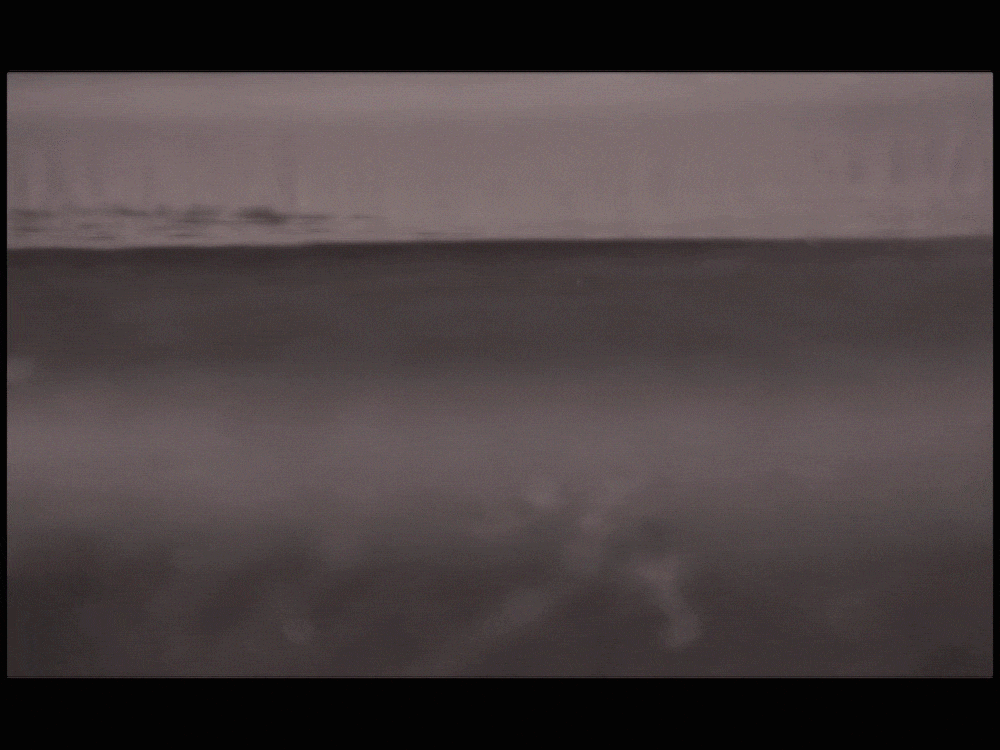

Supplement: Supplementary file 3 — Supplementary file3 (GIF 4757 kb) [file 40820_2021_777_MOESM3_ESM.gif]
